# An Overview of Seasonal Changes in Oxidative Stress and Antioxidant Defence Parameters in Some Invertebrate and Vertebrate Species

**DOI:** 10.1155/2016/6126570

**Published:** 2016-04-04

**Authors:** Gagan Bihari Nityananda Chainy, Biswaranjan Paital, Jagneswar Dandapat

**Affiliations:** ^1^Department of Biotechnology, Utkal University, Bhubaneswar 751004, India; ^2^Department of Zoology, College of Basic Science and Humanities, Orissa University of Agriculture and Technology, Bhubaneswar 751003, India

## Abstract

Antioxidant defence system, a highly conserved biochemical mechanism, protects organisms from harmful effects of reactive oxygen species (ROS), a by-product of metabolism. Both invertebrates and vertebrates are unable to modify environmental physical factors such as photoperiod, temperature, salinity, humidity, oxygen content, and food availability as per their requirement. Therefore, they have evolved mechanisms to modulate their metabolic pathways to cope their physiology with changing environmental challenges for survival. Antioxidant defences are one of such biochemical mechanisms. At low concentration, ROS regulates several physiological processes, whereas at higher concentration they are toxic to organisms because they impair cellular functions by oxidizing biomolecules. Seasonal changes in antioxidant defences make species able to maintain their correct ROS titre to take various physiological functions such as hibernation, aestivation, migration, and reproduction against changing environmental physical parameters. In this paper, we have compiled information available in the literature on seasonal variation in antioxidant defence system in various species of invertebrates and vertebrates. The primary objective was to understand the relationship between varied biological phenomena seen in different animal species and conserved antioxidant defence system with respect to seasons.

## 1. Introduction

Types of seasons and their duration may vary from one ecological region to another and influence the physiology of the inhabiting flora and fauna [1]. Variations in ecological factors such as temperature, duration of sun light exposure, humidity, rainfall, and oxygen content and salinity in aquatic bodies are attributed to observed seasonal physiological changes. Several important physiological responses in animals such as reproduction, hibernation, aestivation, immune functions, behaviour, and susceptibility to various diseases are greatly influenced by two important ecological cues such as day length and temperature [2, 3]. Since all of the above physiological phenomena are manifestation of metabolic status of an animal, any change in seasonal factors will have profound effects on its metabolic activities. This may be one of the reasons to motivate several laboratories to construct a baseline data on physiology of animals with respect to seasons in order to understand the mechanism by which above physiological processes are governed.

Metabolism is a direct reflection of cellular respiration where molecular oxygen is tetraelectronically reduced to water in electron transport chain of mitochondria to generate ATP to meet their energy requirement [4]. Nevertheless, reactive oxygen species (ROS) such as superoxide radicals, hydroxyl radicals, and hydrogen peroxide are generated due to incomplete reduction of molecular oxygen in electron transport chain of mitochondria [5] and as by-products of other biochemical reactions of metabolism [6]. Although ROS are generally considered harmful to cells, their important contribution in regulating various important physiological processes is gradually gaining importance and is reviewed in detail [6, 7]. Involvement of ROS in signal transduction [8], oxygen sensing [9], immune system [10], and regulation of gene expression [11] and in regulating cell functions [12] is well established. Further protection of biological molecules from harmful effects of various external agents by antioxidant defences of cells is well documented [13].

There are several reports in literature which have clearly demonstrated alterations in concentrations of various pollutants in air and water with respect to seasons [14, 15]. Generally, such agents enhance oxidative stress (OS) [16, 17]. Therefore, a change in seasons may influence ROS status and antioxidant defence systems in animals and, thereby, their physiology. The primary objective of the present review article is to string together the pieces of information available in literature on seasonal variation in antioxidant defence system in invertebrate and vertebrate species to have comprehensive information in one platform.

## 2. Antioxidant Defences and Oxidative Stress

Being highly reactive and nonspecific in nature, ROS usually oxidize biomolecules such as lipids, carbohydrates, proteins, and DNA and, thereby, impair normal cellular functions. A shift in balance between oxidants to antioxidants in favour of oxidants is termed as “oxidative stress.” Oxidative stress is considered as cause or effects of several pathophysiological conditions, diseases, and aging processes [17].

**Figure 1:**
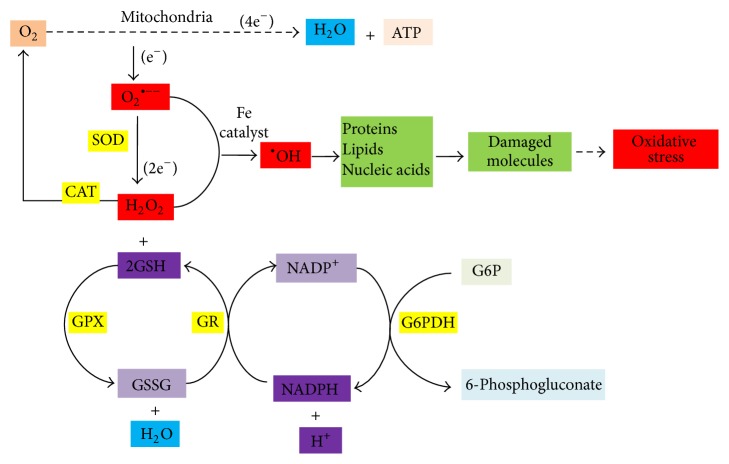
Schematic representation of cellular production of ROS, their impact on biomolecules, and their metabolism by antioxidant enzymes. SOD, superoxide dismutase; CAT, catalase; GPX, glutathione peroxidase; GR, glutathione reductase; O_2_^•−^, superoxide radicals; GSH, reduced glutathione; GSSG, oxidized glutathione.

Antioxidant defence system comprises both enzymatic and nonenzymatic components. Enzymatic system contains a cascade of enzymes which are together known as antioxidant enzymes (AOE). Antioxidant enzymes are ubiquitous and highly conserved in their catalytic nature. Some of them are present in multiple forms. The first member of this cascade is superoxide dismutase (SOD) which dismutates O_2_^•−^ to H_2_O_2_. Hydrogen peroxide is neutralized by two enzymes. One of them is catalase (CAT) and the other one is glutathione peroxidase (GPx) [17]. Catalase breaks down H_2_O_2_ to oxygen and water, whereas GPx reduces H_2_O_2_ and organic hydroperoxides by coupling them with oxidation of reduced glutathione (GSH). Glutathione reductase (GR) plays a major role in generating reduced glutathione from oxidized glutathione by oxidizing NADPH. Subsequently, NADPH is generated from NADP by the enzyme glucose-6-phosphate dehydrogenase (G6PDH) (Figure 1). SOD is of three types depending upon the prosthetic group they carry. They are Fe-SOD, Mn-SOD, and Cu-Zn SOD. Fe-SOD is usually found in bacteria. Cu-Zn SOD is mainly found in the cytoplasm, whereas Mn-SOD is exclusively mitochondrial in nature. Besides, another type of Cu-Zn SOD is reported in extracellular space and is known as EC-SOD [17]. GPx has several isoenzyme forms. GPx primarily functions to detoxify low levels of H_2_O_2_ in cells, whereas CAT assumes more significance in protecting against severe oxidant stress [18]. Nonenzymatic defence system comprises small organic molecules that scavenge various ROS. They are polyphenols, ascorbic acid, tocopherol, carotenoids, reduced glutathione, and so forth [17].

## 3. Seasons and Metabolism

Animals have developed various physiological strategies such as hibernation, aestivation, and migration because they do not have control over the physical components of the environment such as food availability, temperature, and day length [19]. Since all such physiological strategies are directly related to their metabolism the possibilities of generation of ROS and their neutralization with seasons cannot be ignored.

The central focus of this mini review is to compile pieces of information available in literature on various parameters related to ROS and antioxidant defences in some vertebrates and invertebrates of different phyla with respect to seasons under one platform. Since components of antioxidant defence system are highly conservative, it will be interesting to find out how different species manipulate their antioxidant defences to adjust themselves to seasons.

## 4. Seasonal Changes in Antioxidant Defence Parameters in Some Invertebrate and Vertebrate Species

### 4.1. Invertebrates

Metabolic activities and mitochondrial functions of invertebrates are regulated by several components of seasons such as photoperiod, temperature, humidity, and food availability [20–22]. Since metabolic activities in invertebrates change as a function of seasons, it is pertinent to assume occurrence of seasonal changes in their antioxidant defence parameters. Seasonal variation in antioxidant defence and oxidative stress parameters of invertebrates particularly in coastal or marine region are performed by several workers to develop suitable biochemical markers to monitor pollution [16, 23]. The general approach is to compare antioxidant parameters of tissues of a species with polluted and nonpolluted environments and extrapolation of the data to oxidative damage occurring in the tissue. For the purpose, various invertebrate species of different phyla were preferred. Particularly, sessile species are preferred because they do not change their geographical location frequently during investigation.

However, the major limitation in the use of antioxidant molecules or OS parameters as biomarkers to monitor environmental pollution is the lack of basic information on their seasonal variation. It is essential to ascertain the normal seasonal changes in antioxidant defence parameters before using them to monitor environment contamination. The lack of systematic information on the responses of antioxidant defences and OS markers of species to pollutants makes it difficult to use them as biomarkers to assess pollution.

#### 4.1.1. Annelida

Annelids (phylum Annelida) include ringworms, earthworms, and leeches. Annelids have adapted to various ecological zones which span aquatic (both marine and freshwater environments) to terrestrial environment. Seasonal variation of antioxidant defence system in species of this phylum is scanty.* Laeonereis acuta*, a polychaete, is commonly found in estuaries. The species is also used as biomarkers for pollution studies. Antioxidant enzymes such as SOD and CAT and OS parameter such as lipid peroxidation were investigated in the species with respect to seasons [24]. A higher CAT activity was recorded in autumn than in other seasons; lipid peroxidation did not exhibit seasonal variation [24]. It indicates that the species can modulate its redox response seasonally to keep OS level of its tissue unchangeable. Earthworms are used to increase soil fertility. No information is available on seasonal variation in antioxidant defence system of earthworms. Whether such variations in antioxidant parameters have any impact on health status of earthworms and/or their efficiency to increase soil fertility deserves special attention.

#### 4.1.2. Mollusca

Molluscs (phylum Mollusca) encompass a large number of species that spread from aquatic to terrestrial ecosystem. Impacts of tidal heights on activities of antioxidant enzymes in gills and digestive glands of* Mytilus edulis* inhabiting same intertidal zone of France in two consecutive autumns were reported [25]. Results have shown that activities of SOD, CAT, GPx, and GR were comparatively higher in both the tissues obtained from the species inhabiting high shore compared to low shore. It is to note that exposure of* Mytilus* to air was more (6 h/12 h) at high shore than that at low shore (2 h/12 h). The authors cautioned that for ecotoxicological studies due importance must be given on sampling sites [25].

It was reported that DNA strand fragmentation was elevated in tissues of* Mytilus galloprovincialis* collected from an unpolluted site in Adriatic Sea of the species in October which was accompanied with an increase in CAT activity and LPx level [26]. The authors also noticed that DNA fragmentation in tissues of the species was considerably influenced by the concentration of trace metals present in the water samples [26]. Activities of antioxidant enzymes such as SOD and CAT as well as the level of LPx in gills and mantle tissues of* Mytilus galloprovincialis* were measured to monitor heavy metal pollution of the water of the Saronikos Gulf of Greece [27]. The authors restricted their investigation to three polluted sites in that area along with one unpolluted site. On the basis of their three-year repeated study, they opined that seasonal variation in activities of antioxidant enzymes and the level of LPx, an index of OS, can be used as potential biomarkers of toxicity for long-term monitoring of marine coastal ecosystems [27]. A careful observation of data presented by the above authors suggests that activity of SOD and CAT and level of LPx in tissues of mussel from control sites were higher in winter in comparison to the other two seasons such as spring and summer. A clear and repeatable pattern of seasonal variations in above parameters was noticed from their study. It was observed that SOD activity was higher in summer than in other seasons, while CAT activity was higher in winter and spring than in summer in polluted areas.

Antioxidant defence system in oyster (*Crassostrea rhizophorae*) also exhibits seasonal variation. Total oxygen radical scavenging capacity, level of LPx, and activity of GST in gills of immature adult mangrove oysters collected from polluted and nonpolluted sites of Brazilian coast were investigated in detail by Zanette et al. [28]. The authors had the belief that temperature does not play a detrimental role in seasonal variation of antioxidant defence parameters in oysters. They concluded that the observed seasonal changes in activities of CAT and GST in tissues of oysters were due to the geographical location of the sampling site.

Lack of water, humidity, and food in a specific season leads to metabolic depression in snails. In summer, they undergo aestivation, whereas in winter they undergo hibernation. After arrival of suitable environment conditions, the animal arouse from the metabolic depression. In general, aestivation followed by arousal is accompanied by a transient elevation in oxygen consumption leading to elevated ROS production [29, 30]. Several studies in land snails as well as water snails have shown augmentation of certain redox regulatory molecules in their tissues [31, 32]. It was noticed that the pattern of OS markers and levels of various antioxidant enzymes were different in tissues of* Helix aspersa* in aestivation followed by arousal. And the pattern of data was dependent on the seasons [32]. The authors noticed that changes in levels of antioxidant enzymes not only were influenced by seasons but also were tissue specific. On the basis of their findings, the authors proposed the presence of internal clock in tissues of land snail,* Helix aspersa*, which controls the free radical metabolism in different seasons.

Due to the sedentary nature, sessile-filter feeding, wide geographical distribution, and abundant availability,* Perna viridis*, a bivalve mollusc, is preferred by ecophysiologist to use it as a marker species for pollution detection in coastal water. Detailed investigations on seasonal changes in antioxidant enzymes (SOD, CAT, GPx, GST, and GR), small antioxidants (ascorbic acid and GSH), and OS markers (LPx and H_2_O_2_) were carried out in gills and digestive glands of natural population of green lipped mussel (*Perna viridis*) in Bambolim beach area of Goa, India [33]. The authors noticed two important aspects regarding the antioxidant defence status in tissues of the species. Firstly, antioxidant defence status of tissues of* P. viridis* exhibited seasonal variation and, secondly, components of antioxidant capacity of tissues with respect to seasons varied from each other. Considering antioxidant defence parameters, both enzymatic and nonenzymatic components, the authors were of the opinion that changing environmental factors during seasons have crucial impact on regulating antioxidant defence mechanism in this species.

#### 4.1.3. Arthropoda

Most arthropod species are land dwellers and few are aquatic in nature. Niyogi et al. [34] studied the seasonal variation of antioxidant enzymes in barnacle (*Balanus balanoides*). They are dominant species in rocky shores. The authors noticed high levels of antioxidant enzymes and GST in digestive gland of the species in premonsoon period or summer followed by gradual decrease in their activities in monsoon with minimum levels in winter. Microsomal LPx exhibited a reverse trend of seasonal variation to that of antioxidant enzymes. Also the authors noticed a positive correlation between PAH and levels of antioxidant enzymes in the tissue.

A study on different antioxidant enzymes in honey bee (*Apis mellifera*) revealed that levels of antioxidant enzymes and peroxides were higher in active season (August-September) in comparison to the other months [35]. The authors suggested that elevated levels of antioxidant enzymes and oxidants in honey bee during active months may be due to their elevated activity to collect nectar. High flight activity of bees in months of August and September leads to a higher demand of oxygen which may cause higher production of ROS in active seasons. It was noticed that various components of antioxidant defence system in tissues of mud crabs (*Scylla serrata*) exhibited seasonal changes that are tissue as well as gender specific [36]. Also the authors recorded an elevation of OS markers in summer season, when the temperature and salinity of the lagoon were higher than those in other seasons.

#### 4.1.4. Echinodermata

All species of the phylum are marine dwellers. A seasonal variation in feed intake under constant temperature and natural photoperiod was noticed in green sea urchin [37]. In another species,* Loxechinus albus*, higher contents of ascorbyl radical and alpha tocopherol in gonads were recorded in winter than in summer and spring seasons [38].

Taken together, the above discussion reveals that invertebrates adapt different strategies to regulate their antioxidant defences in response to seasonal factors depending on ecological demands/environment.

### 4.2. Vertebrates

Season-related changes in antioxidant defence parameters in case of vertebrates indicate a composite reflection of impacts of several biotic and abiotic factors. Biotic factors mainly include migration, reproduction, metabolic status, and age of animals, while abiotic factors are mainly temperature, photoperiod, salinity, oxygen content, food availability, and so forth.

#### 4.2.1. Pisces

Literature reveals that most of the studies related to seasonal variation of antioxidant defences in fish are investigated with respect to pollutants present in water. A decrease in GSH content and an increase in GR activity in hepatopancreas of kill fish population (*Fundulus hetroklitus*) collected from creosote inlet of Elizabeth River were noticed from early to late summer [39]. Gorbi et al. [40] compared responses of several antioxidant defence parameters such as CAT, GPx, and GSH and total antioxidant capacity in tissues of two species of fish,* Anguilla anguilla* (European eel) and* Mugil cephalus* (stripped mullet), in Mediterranean lagoons in different seasons. Although responses of antioxidant defence parameters of fishes were influenced by temperature and reproductive cycle, the pattern of responses was species specific. Unlike striped mullet, antioxidant defence parameters of eels were independent of sexual maturation but were markedly influenced by high temperature of summer.

A comparative study on seasonal variation in antioxidant defence parameters and OS indices in tissues of four species of fish (*Micropogonias furnieri*,* Pimelodus pintado*,* Loricariichthys anus*, and* Parapimelodus nigribarbis*) from Southern Brazil suggested that changes in antioxidant enzymes with relation to season were not only species specific but also tissue specific [41]. Radovanović et al. [42] reported that the level of SOD was higher in liver and muscle of barbel (*Barbus barbus*) in spring than in summer but CAT activity was found to be in reverse order. It was reported that levels of CAT and GR enzymes in liver of Senegalese sole (*Solea senegalensis*) exhibited seasonal variation [43].

#### 4.2.2. Amphibia

Although amphibians are very important group used for study as bioindicators of environmental pollutions, seasonal studies related to antioxidant defences were limited to few species. An elaborate investigation has noticed the occurrence of seasonal fluctuation of antioxidant proteins in liver tissues of* Rana ridibunda* [44]. The authors registered a collapse of antioxidant defence system in summer in the species in comparison to the other seasons. A seasonal variation of the apoptotic and antioxidant proteins in the heart and muscle of water frog* Pelophylax ridibundus* was recorded [45]. The authors noticed that, during overwintering, levels of antioxidant enzymes such as SOD, CAT, and GPx were low due to metabolic depression and recovery from overwintering resulted in increase in production of antioxidant enzymes to overcome OS as the consequences of arousal.

#### 4.2.3. Reptilia

Not much information is available on free radical metabolism in reptiles in general and snakes and crocodiles in particular with reference to seasons. Reptiles are poikilotherms. External environmental factors have profound impacts on metabolism of reptiles in general and free radical metabolism in particular. Reptiles have adapted to various ecological niches which span from completely dry atmosphere of desert to submerged aquatic habitat and some of them make long distance movement. Though there are few reports on free radical metabolism in reptiles, not much information is available in relation to seasonal variation. In this regard, crocodiles deserve special attention because submerged habitat had made crocodiles with more tolerance to hypoxia, since hypoxia is directly related to redox metabolism; crocodiles are an excellent model to establish relationship between hypoxia and OS.

A preliminary study from Hermes-Lima's laboratory [46] for the first time demonstrated a relationship between developmental stages in crocodile and antioxidant defence system. An increase in oxidative damage was recorded in tissues of juveniles during winter to summer transition. The changes in GSSG and thiobarbituric acid reactive substances (a form of LPx) in tissues of juvenile crocodiles in summer were attributed to differences in nocturnal temperatures of their habitat in winter and summer seasons [47]. Not much information is available on seasonal variation of antioxidant defence system in turtles and tortoises. A report from Pérez-Pinzón and Rice [48] revealed that ascorbic acid and reduced glutathione contents undergo seasonal variation in central nervous system of anoxia-tolerant turtles. The authors opined that seasonal variations in antioxidants had some contribution in tolerating the hypoxia-reoxygenation in turtles, a characteristic of diving behavior.

#### 4.2.4. Aves

Birds are homeotherms. Some reports indicate occurrence of seasonal variation in their antioxidant defence system and OS status. Habitat and photoperiodism may be responsible for the observed seasonal variation of OS metabolism in their tissues. Poor habitat due to unavailability of food may induce intensive foraging activity which may increase generation of ROS in birds. In case of Seychelles warblers (*Acrocephalus sechellensis*), a seasonal variation in OS in terms of generation of ROS was attributed to low availability of food [49]. A detailed study on seasonal variation of different OS physiology parameters was investigated in barn swallow (*Hirundo rustica*) by Raja-aho et al. [50]. It was noticed that wintering birds had high biotransforming activities with higher OS level in comparison to spring and summer birds. Such variation in biotransforming enzymes in birds may be associated with their preparation for migration. Such phenomena are now accepted as “preparation for oxidative stress” [51].

#### 4.2.5. Mammals

Mammals inhabiting temperate zone usually exhibit seasonal changes in their morphology, behavior, and physiology in order to cope with changing environment. Therefore, they can exhibit seasonal variation in their antioxidant defence system and OS status. Photoperiod has been shown to affect lipid droplets and LPx in bank vole [52]. Several studies have shown that physiology of bovines (cows and buffalos) is significantly affected by the higher temperature of summer. This phenomenon is known as summer heat stress which influences feed intake and milk production in case of bovines. Studies have demonstrated that summer heat stress was accompanied with elevated OS markers and decrease in antioxidant enzymes [53–55]. It was reported that high ambient temperature during summer was responsible for the increase in both body temperature and OS and also reduction in estrus signs in Japanese cow [56]. The pioneer work of Majić-Balić et al. [57] demonstrated that semen quality of young bulls deteriorates due to intensive prooxidative effects in seminal plasma during summer season. Rats exposed to high temperature in summer had higher OS and low levels of antioxidant enzymes in their erythrocytes in comparison to winter season [58]. One of the interesting observations was the increase in the observed LPx level in hearts of patients, rats, and guinea pigs during summer compared to winter [59–62].

## 5. Conclusion

**Figure 2:**
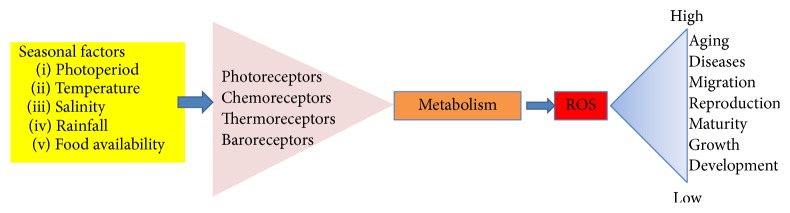
A schematic figure showing how seasonal factors affect various physiological functions through reactive oxygen species.

In the present review an attempt is made to summarize information available in the literature on seasonal changes in antioxidant defence system and OS markers in invertebrates as well as in vertebrates. It is inferred that seasonal variation in antioxidant defence system may be an evolutionary strategy by animals for different adaptations against various physical aspects of environment. Also a far-fetched implication of changes in seasonal factors on global food chain cannot be denied as larval or embryonic forms of invertebrates constitute first line of primary productivity. It is apparent that various cues of seasons may regulate metabolic functions in tissues through appropriate receptors which in turn decide ROS and antioxidant defences in tissues and, thereby, govern various physiological activities in the animals (Figure 2).
